# Here, the huge rainbow within the COVID-19 storm

**DOI:** 10.1016/j.eclinm.2020.100654

**Published:** 2020-11-20

**Authors:** Ilaria Capua, Mario Rasetti

**Affiliations:** a One Health Center of Excellence, University of Florida, McCarty Hall D, 1604 McCarty Dr, Gainesville, FL 32611, USA; b Institute for Scientific Interchange (ISI) Foundation, Via Chisola 5, 10126, Turin, Italy

The real-life experience of the 2020 pandemic has shown us how totally unprepared we were to face such an event, albeit an event with several unique features. The inter-species jump by an animal virus to humans is, perhaps, one of its least unique characteristics as this has occurred several times in the past. The total absence of pre-existing immunity in the human host was the worst possible scenario in the face of rapid worldwide virus dissemination through international travel making COVID-19 effectively unstoppable. Further, this pandemic overlies other epidemics of non-communicable diseases (NCDs) such as cancer, obesity and chronic respiratory diseases, supporting a novel proposal for the term “syndemic” [1]. But there is more: this virus is also spilling into animal populations such as mustelids which could become reservoirs of infection, thus potentially allowing the evolution of many host-adapted lineages [2]. This observation leads us to consider another term which unveils another unique feature: its panzootic nature [3]. Panzootic refers to “all animals” and should taxonomically include *Homo sapiens*. This concept, of a virus capable of infecting multiple animal species, provides a further perspective to this epochal event.

Several animal viruses have jumped the species barrier to humans, with varying degrees of success. This evidence combined with the many interlocking aspects of emerging and re-emerging zoonotic diseases, lies at the core of what we know as the One Health approach [4,5]. This approach has generated extensive literature on how infectious diseases should be managed with particular focus on areas where the human/animal/environmental domains overlap. Studies developed over decades have allowed the identification of hotspots and high-risk events of potential pathogen emergence. Generally, human invasion of segregated areas, the presence of live animal markets in large cities in combination with poor healthcare services and insufficient surveillance activities at the human-animal interface provides a fertile ground for pathogens to cross the species barrier. The One Health subject matter created over the last 40 years around the human/animal/environment interface should now be fully recognized as a model and become the interdisciplinary heart of a new paradigm shift.

The reason for this is that COVID-19 demonstrated that a human pandemic may be predictably linked to the spillover of an animal virus, but its course and ramification are linked to multiple other drivers such NCDs, poverty, mobility and of course, political will. Thus, the current pandemic acts as a multi-system stress test for interconnected and interdependent domains. Not only it impacts health and healthcare systems directly; it adds complications to the management of personal interactions and mobility patterns causing us to change many of our normal social behaviors. In addition, the effects of COVID-19 have been more devastating in certain geographical and cities that their permanent transformation seems inevitable. In a time in which we will be forced to rethink entire systems which have been disrupted by the pandemic we should be able to develop convergence mechanisms for human, animal, plant, and environmental health dovetailing into the UNs Sustainable Development Goals [6,7].

This approach would be totally unrealistic if COVID-19 had not occurred in 2020. Among its unique characteristics is that this pandemic has come in the digital era. Virtually every variable of the pandemic is being measured in some form. In addition to biomedical data, other data is actively and passively generated by the billions of personal or institutional devices we communicate with and from. Weather data on allergen concentration, pollution levels, and many more variables, such as mobility and all the features of everyday life that favor contagion, can be explored as drivers of hospitalizations and excess deaths. COVID-19 is the perfect storm to maximize the potential of the data that we are generating in real time to support data-driven decision making in the short and medium term. Artificial Intelligence is the tool to extract information from the correlations within such diverse data sets, and create the relevant scenarios [8,9].

In 2006 we were faced with similar real-world One Health question: should genetic sequences of zoonotic pathogens be shared across disciplines in publicly accessible databases. The need for a broader and “open” informatic infrastructure to collect, share and study genetic information on pre-pandemic viruses was established at the height of the H5N1 “Bird Flu” crisis [10]. The platforms and bylaws created during that emergency have revealed themselves as the essential repositories to collect and analyze hundreds of thousands of COVID-19 genomes in 2020–14 years after they were conceived. Today COVID-19 research benefits from the foundational concepts created at that time.

Crises cannot be wasted and must become opportunities. Here, the huge rainbow embedded in the COVID-19 storm is that it offers us the opportunity to explore the development of a multi-centric informatic networked infrastructure to host and analyze large sets of heterogeneous data. We need to aim at interrogating these data using an integrated approach to generate a pathway towards the full understanding of the multifaceted implications of the current event in all its magnitude. We have the tools to understand whether and when correlations imply causation, and thus acquire the wisdom necessary to govern the process.

Without a doubt, COVID-19 is more than just a pandemic with dire consequences: it is an accelerator of interdisciplinarity, an opportunity to change for the better, and a real-time example of why we need to capitalize on our existing knowledge to discover novel converging forces. Major questions lie ahead as the disruption caused by COVID-19 unfolds. Convergence, interdisciplinarity and a novel and circular approach which aims at co-advancing the health of humans, animals, plants, and the environment can be the starting point of the rainbow. New, brave and transformational synergies must be explored to redesign a health eco-system which is less vulnerable and truly more sustainable in the face of this syndemic, which has been triggered by a pandemic-, and which may well evolve into a panzootic.

## Author contribution

All authors contributed equally to the manuscript.

## Declaration of Competing Interest

The authors declare that there is no conflict of interest in the writing of this commentary.
